# Unveiling the Anti-Inflammatory Effects of Antidepressants: A Systematic Review of Human Studies over the Last Decade

**DOI:** 10.3390/ph18060867

**Published:** 2025-06-10

**Authors:** Layla Bleibel, Paulina Sokołowska, Gabriela Henrykowska, Jacek Owczarek, Anna Wiktorowska-Owczarek

**Affiliations:** 1 Department of Pharmacology and Toxicology, Medical University of Lodz, Żeligowskiego 7/9, 90-752 Lodz, Poland; paulina.sokolowska@umed.lodz.pl; 2 Department of Epidemiology and Public Health, Medical University of Lodz, Żeligowskiego 7/9, 90-752 Lodz, Poland; gabriela.henrykowska@umed.lodz.pl; 3 Department of Hospital Pharmacy, Medical University of Lodz, Muszyńskiego 1, 90-151 Lodz, Poland; jacek.owczarek@umed.lodz.pl

**Keywords:** inflammation, depression, cytokine, SSRI, SNRI, ketamine

## Abstract

**Background/Objectives**: Depression ranks among the most prevalent mental health conditions globally, marked by a variety of symptoms that frequently cause significant emotional distress and impairment in individuals, alongside a high recurrence rate. The predominant approach to treating depression revolves around monoamine theory, utilizing SSRIs and SNRIs, with Esketamine emerging as a supplementary option in recent times. Nevertheless, there is a growing focus on exploring the relationship between inflammation and depression, revealing a strong correlation between the two. This insight prompts consideration of the anti-inflammatory properties of current antidepressants in their therapeutic application. **Methods**: A systematic literature search was conducted using the PubMed database to identify randomized controlled trials (RCTs) and clinical trials (CTs) that assessed the in vivo anti-inflammatory effects of SSRIs (fluoxetine, escitalopram, sertraline, and paroxetine), the SNRI venlafaxine, and esketamine/ketamine in human subjects undergoing treatment for depression. The included studies were evaluated based on changes in levels of pro-inflammatory and anti-inflammatory markers in response to the antidepressant treatments. **Results**: SSRIs, SNRIs, esketamine, and ketamine (a racemic mixture of S- and R-ketamine not formally approved for the treatment of depression) exhibit anti-inflammatory effects through diverse mechanisms, such as reducing pro-inflammatory cytokines or enhancing anti-inflammatory cytokines in serum or within specific brain regions like the hippocampus and prefrontal cortex. These actions are mediated through various inflammatory pathways, including nuclear factor kappa-light-chain-enhancer of activated B cells (NF-κB), the brain Nod-like receptor pyrin-containing 3 (NLRP3) inflammasome, the glutamatergic system, the gut–brain axis, the hypothalamic–pituitary axis, impaired neuroplasticity, and the kynurenine pathway. **Conclusions**: In summary, SSRIs, SNRIs, esketamine, and ketamine exert an anti-inflammatory role alongside their antidepressant effects via these intricate mechanisms.

## 1. Introduction

Major depressive disorder (MDD), commonly referred to as depression, stands as the most prevalent and highly debilitating mental health condition worldwide, affecting 3.8% of the global population, with a notable impact on 5% of adults, as highlighted by the World Health Organization [1]. This disorder takes a significant socioeconomic toll, with only a fraction—approximately 30% to 35%—of affected adults achieving remission through current therapeutic approaches [2,3,4]. Moreover, the recurrence rate following the initial major depressive episode is as high as 60% [5]. Projections estimate that by 2030, depression will be the second most serious global health condition [6]. Furthermore, after the coronavirus disease (COVID) in 2020, cases of major depression increased by more than a quarter (28%) worldwide [7].

MDD pathogenesis is complex and most likely multi-factorial with various mechanisms interacting and affecting one another. Several hypotheses have been proposed to explain its pathogenesis, including the monoamine hypothesis, the cytokine hypothesis, the neuroplasticity hypothesis, and the hypothalamic–pituitary–adrenal (HPA) axis hypothesis [8,9,10,11].

The primary treatment of depression is derived from the monoamine hypothesis. Following the incidental discovery that two tuberculosis drugs alleviated the symptoms of depression, the monoamine theory of depression was born, around 50 years ago [12,13]. This theory hypothesized that the deficiency of noradrenergic and or/serotonergic neurotransmitters is the basis of the cause of depression, and replenishing these neurotransmitters can act as a treatment for depression [14,15].

In the late 1960s, the mechanisms of serotonin degradation, synthesis, and reuptake were being characterized. Then, in the early 1970s, based on trying to increase serotonin neurotransmission in the synaptic cleft, the first serotonin reuptake inhibitor (SSRI) fluoxetine was introduced [16,17]. Clinical trials lasted more than 7 years, although it was approved by the US Food and Drug Administration (FDA) in 1987 [18]. The first serotonin–norepinephrine reuptake inhibitor (SNRI), venlafaxine, was introduced in 1993 in the United States market [19].

However, recent studies have suggested that the monoamine hypothesis of depression should be revised, as the pathogenesis of MDD is still unclear and it does not provide a clear explanation of the action of antidepressants [15]. Moreover, they are effective in less than 50% of patients [20], and require chronic treatment to induce an effect [21,22]. The revised monoamine hypothesis suggests that monoamines play a modulatory role on other neurobiological systems that have a more primary role in depression or that they must be present in the context of stressors [23,24].

Clinical research has uncovered evidence indicating dysfunction in the glutamatergic system among patients with MDD. This includes findings such as increased concentrations of glutamate in plasma [25,26,27] and increased concentrations of glutamine in cerebrospinal fluid (CSF) [28]. Ketamine, originally developed as a dissociative anesthetic in the 1960s and FDA-approved in 1970 [29], is a noncompetitive N-methyl-D-aspartate (NMDA) receptor antagonist [30,31,32]. Additionally, it exhibits weak binding to sigma [33], muscarinic [34], and κ and δ opioid receptors [35], as well as to dopamine, norepinephrine, and serotonin transporters [36]. Ketamine blocks NMDA receptors, leading to calcium influx and the subsequent release of brain-derived neurotrophic factor (BDNF). This release activates pathways that promote synaptogenesis and neurogenesis, ultimately reducing depressive symptoms [37].

Overall, clinical data suggests that the glutamatergic system plays a role in the pathophysiology of MDD, involving disruptions in glutamatergic substrate concentrations and alterations in NMDA receptors [38,39,40,41]. Recent clinical breakthroughs have led to the FDA approval of esketamine, the S-enantiomer of ketamine, as a nasal spray for adjunctive treatment in patients with treatment-resistant depression, signifying the growing recognition of its therapeutic potential. However, it is important to emphasize that this approval applies exclusively to esketamine, not to racemic ketamine. In our current study, we examine the effects of both compounds [42] because ketamine has been widely studied for its anti-inflammatory and antidepressant properties.

Given the dominating monoamine theory of depression and the widespread use of SSRIs and SNRIs as primary treatments, along with esketamine’s role in treating treatment-resistant depression, it is crucial to explore their potential anti-inflammatory effects. This is especially important as emerging theories, such as the link between inflammation and depression, are gaining attention [43]. Understanding how antidepressants influence inflammatory pathways, particularly their effects on pro- and anti-inflammatory cytokines, may offer valuable insights into their therapeutic mechanisms. The aim of this systematic review is to evaluate the anti-inflammatory effects of antidepressants from the SSRI, SNRI, and esketamine (including ketamine) classes, specifically focusing on their impact on cytokine levels as a marker of the inflammatory response. This review will analyze human studies that have quantitatively assessed changes in cytokine levels—whether an increase, decrease, or no effect—in response to antidepressant administration.

## 2. Relationship Between Inflammation and Depression

Before exploring the link between antidepressants and inflammation, it is essential to first examine the connection between inflammation and the pathogenesis of depression. Infection, chronic stress, and stress-inducing events that cause a systemic inflammatory response are widely recognized as significant contributors to the pathogenesis of depression [44,45]. The inflammatory process and immune system activation in depression have been observed in both the peripheral and central nervous systems (CNS). More specifically, communication between peripheral inflammation and the CNS was identified to be mediated mainly by the nuclear factor (NF-κB) at the blood–brain barrier [46,47]. Interleukine1 beta (IL-1β) [48], Tumor Necrosis Factor Alpha (TNF-α) [49], and Interleukine1 (IL-1) are the primary systemic inflammatory cytokines inducing the body’s inflammatory response and serve as important biomarkers of inflammation [11]. TNF-α release into the bloodstream initiates a cascade of immune system activation and release of additional pro-inflammatory cytokines such as IL-8, IL-1β, and IL-6 [50]. This systemic inflammatory response leads to a disruption of the blood–brain barrier (BBB), a highly selective and semi-permeable membrane that protects the brain and CNS from pathogens. However, systemic inflammation causes a disruption in the integrity and permeability of the membrane which allows inflammatory mediators to reach the brain and trigger a neuroinflammatory reaction [51].

**Figure 1:**
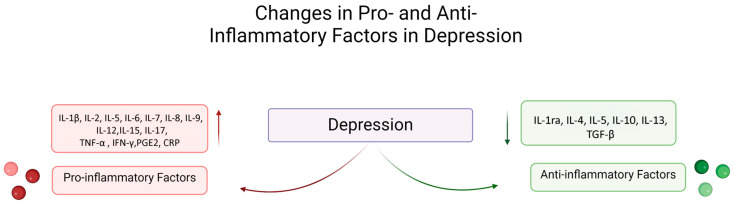
Changes in the main pro- and anti-inflammatory factors in depression. The red arrow represents pro-inflammatory factors and their increase in depression, while the green arrow represents anti-inflammatory factors and their decrease in depression. Created with Biorender.

The association between inflammation and depression was initially discovered when multiple studies found that administering IL-2 and IFN-gamma to patients triggered depressive symptoms such as anhedonia and fatigue [52,53,54,55,56,57,58]. Elevated levels of pro-inflammatory cytokines were found in patients with MDD, even though they had no underlying somatic disease [59] (Figure 1). Furthermore, growing evidence suggests that individuals with depression also exhibit elevated levels of circulating cytokines like IL-6 [49,60,61], IL-1β [48], TNF-α [49], IFN-α, prostaglandin E2 (PGE2), and chemokine CCL2 [49,62,63,64,65], as well as acute-phase proteins such as C-reactive protein (CRP) [66]. This relationship has also been observed in patients with systemic diseases that have an inflammatory pathophysiology such as diabetes mellitus type 1 [67], inflammatory bowel disease, and rheumatoid arthritis [68]. Moreover, reduced levels of the anti-inflammatory cytokines transforming growth factor (TGF-β) and IL-10 were observed in the brain [69]. These factors all indicate the presence of chronic low-grade inflammation in MDD [70]. Patients with higher levels of inflammatory factors have also been correlated with a decreased response to antidepressant treatment [71], precisely SSRIs, and a better response to an SNRI or an add-on regimen with bupropion or anti-inflammatory agents [72,73] (Figure 1).

### 2.1. Mechanisms of Inflammation

Peripheral inflammation can contribute to depressive symptoms by reaching the CNS through various mechanisms, including the gut-microbiota–brain axis, the HPA axis, the glutamatergic system, and the kynurenine (KYN) pathway, as well as by impairing neuroplasticity [74,75,76,77].

Chronic stress leads to the activation of macrophages and monocytes, which in turn trigger the release of pro-inflammatory cytokines such as IL-1β, IL-1, and IL-6 [78,79]. These peripheral pro-inflammatory cytokines can reach the CNS and stimulate glial cells and astrocytes. Through a feedback mechanism, these CNS immune cells then produce cytokines [80], leading to neuroinflammation, which is likely to play a significant role in the development of depressive symptoms [61,81,82]. This process is thought to be mediated by peripheral macrophages, as studies inhibiting these cells have shown reduced levels of pro-inflammatory cytokines [83,84]. This was known as the macrophage theory of depression, and it was first proposed by Smith et al. in 1991 [85].

### 2.2. Neuroinflammation

#### 2.2.1. Microglia

Microglia are the main immune cells in the CNS, and it was found that in models of neurodegeneration, inflammation, and aging, microglia favor inflammation marker activation [86,87,88]. Then, pro-inflammatory mediators were found to be increased in various causes of depression, such as inflammation, gut dysbiosis, or stress, all of which have been associated with persistent activation of microglia [89,90,91,92,93]. Furthermore, in a neuroinflammatory state, microglia exhibit reduced expression of neuroprotective genes like nerve growth factor (NGF), neurotrophins (NTs) 4/5, glial cell-derived neurotrophic factor (GDNF), and brain-derived neurotrophic factor (BDNF). Under non-inflammatory conditions, these genes offer vital trophic support to neurons, and their reduced expression can impair neurogenesis and synaptic plasticity [94], potentially leading to depressive-like symptoms [95]. It has been suggested that alterations in neurotrophins, including the decreased expression of BDNF observed in the brains of patients with MDD, play a key role in the impaired neuroplasticity. Additionally, pro-inflammatory cytokines further impact BDNF levels in the brain [96,97,98,99]. Studies found that the anti-inflammatory agent minocycline has a neuroprotective effect by decreasing the pro-inflammatory proliferation of microglia [100,101,102].

#### 2.2.2. Astrocytes

Similar to microglia, astrocytes contribute to proper neuronal development by releasing growth factors such as BDNF, vascular endothelial growth factor (VEGF), nerve growth factor (NGF), ciliary neurotrophic factor (CNTF), and fibroblast growth factor 2 (FGF2), also known as basic FGF (bFGF) [103]. Moreover, their function may be compromised during an inflammatory state, affecting their ability to maintain CNS homeostasis and consequently impacting neuronal survival [104]. Elevated plasma levels of glial fibrillary acidic protein (GFAP) and S100β, markers of astrocytic activation, have been observed in patients with treatment-resistant depression compared to healthy individuals [105].

#### 2.2.3. Lymphocyte Infiltration

Recent studies have demonstrated that lymphocytic infiltration can occur in the CNS under specific conditions, challenging the long-standing concept of the CNS as an immune-privileged site [106]. A study conducted by Schlaaf et al., which involved mapping T and B lymphocytes across whole-brain sections, identified distinct patterns of neuroinflammation, characterized by increased densities of T and/or B cells, within the white matter, cortical areas, and limbic system in a significant subset of individuals with schizophrenia and mood disorders. These findings lend support to the hypothesis that lymphocyte infiltration occurs at a greater percentage in patients with mood disorders compared to healthy controls. The observed increase in lymphocyte density suggests a compromise of the blood–brain barrier, as under normal physiological conditions, lymphocytes rarely cross this barrier [107].

#### 2.2.4. Neuroplasticity

Neuroplasticity refers to the ability of the CNS to adapt and modify itself in response to various stimuli, by restructuring and/or rewiring its neural connections [108,109,110,111]. Changes in gray matter volume, measurable through MRI in clinical studies, serve as an important indicator of neuroplasticity [111]. Among brain regions, the hippocampus has been the most extensively studied in the context of depression, with evidence suggesting that stress and other adverse stimuli can impair hippocampal plasticity [112,113].

Neuroimaging studies demonstrate reductions in gray matter volume in MDD, particularly in the hippocampus [114,115] and anterior cingulate cortex [116]. Postmortem analyses found further neuronal and glial loss, particularly in individuals with chronic forms of the illness [117,118]. These observed decreases in the volumes of the prefrontal cortex and hippocampus are thought to result from neuronal and glial disruption and atrophy associated with depression [119,120]. These observations support the neuroplasticity hypothesis of MDD, which proposes that impaired neuroplasticity plays a central role in the pathogenesis of depression [121].

Antidepressants have been shown in experimental studies to enhance neuroplasticity, stimulate hippocampal neurogenesis, and mitigate the deleterious effects of stress on the hippocampus [122,123,124,125]. Supporting these findings in humans, postmortem studies have shown that patients with depression receiving antidepressant treatment exhibit greater total dentate granule cell numbers and increased dentate gyrus volume compared to nonmedicated individuals [126]. While these effects appear to be important in certain models of depressive-like behavior, evidence also indicates that antidepressant efficacy may also involve neurogenesis-independent mechanisms [127].

#### 2.2.5. Oxidative Stress

Reactive oxygen species (ROS) play an important role in not only the pathogenesis of neurological disease but also in normal brain function [128]. Oxidative stress (OS) is characterized by increased production of ROS (free radicals) in the cells and tissues and simultaneously the exhaustion of antioxidative defenses, which causes an imbalance and inability to neutralize ROS. This leads to damage to DNA, lipids and proteins [129,130]. The brain is particularly susceptible to ROS-induced injury due to its high oxygen metabolism and relatively weak antioxidant defenses. Consequently, ROS produced by microglia and astrocytes in brain tissue can trigger neuroinflammation, cell death, and subsequent neurodegeneration and memory loss [131]. Furthermore, ROS-mediated lipid peroxidation in the brain leads to the generation of toxic compounds that induce neuronal cell death [132], representing a significant contributing factor to the pathogenesis of MDD [133].

Therefore, an increase in ROS production and a decreased antioxidant response lead to neurodegeneration, inflammation, tissue damage, and cell death [134]. These processes have been linked to the pathogenesis and progression of MDD [135,136] and it is known as the ‘oxidative stress hypothesis of depressive disorders’ [137,138,139]. Increased ROS levels were also found to modulate feedback in the HPA axis and to effect serotonergic and GABA transmission in experimental rodent models of anxiety and depression [140]. OS is then increased by HPA axis activation [141].

#### 2.2.6. Hypothalamic–Adrenal Axis

Approximately 40% to 60% of patients with MDD experience disturbances in the HPA axis, such as hypercortisolism, mirroring the effects of chronic stress [142]. This can be triggered by HPA axis activation due to increased cytokine production, which can also lead to glucocorticoid receptor resistance [143]. This stress and HPA axis dysregulation result in functional changes not only in the hippocampus [144] but also in the amygdala and prefrontal cortex [145,146]. Stress increases the release of adrenocorticotropic hormone (ACTH), corticotropin-releasing factor (CRF), and corticosterone (CORT) in rats [147]. Increased levels of CORT have also been found to increase NLRP-3 inflammasome levels in the hippocampus, stimulating the pro-inflammatory response [148,149]. Furthermore, increased levels of glucocorticoids for a long period of time also lead to neuronal death, synapse loss, and changes in neuronal dendrites [150]. Additionally, suppression of the HPA axis was seen in a study of patients with depression being treated with antidepressants, decreasing the stress response [151,152].

#### 2.2.7. The Kynurenine Pathway

The kynurenine pathway is intricately regulated by the immune system and has a significant connection to the central nervous system (CNS). Within this pathway, two mechanisms are believed to contribute to inflammation. The first is the tryptophan starvation hypothesis, which suggests that activation of the kynurenine pathway decreases levels of tryptophan as it is converted to kynurenine, which is mediated by indoleamine 2,3-dioxygenase (IDO) and tryptophan 2,3-dioxygenase (TDO) [153], reducing its availability for serotonin synthesis. The second mechanism involves a shift within the kynurenine metabolism pathway toward producing neurotoxic metabolites, particularly quinolinic acid [154]. Quinolinic acid, generated primarily by activated monocytic cells, induces excitotoxicity, stimulates the production of reactive oxygen species (ROS), increases lipid peroxidation in the CNS, and disrupts glutamatergic neurotransmission. This disruption occurs as kynurenic acid and NMDA receptors block glutamate receptors, therefore contributing to excitotoxicity, neuroinflammation, and oxidative stress [155,156,157,158,159]. This is supported by a study on patients with major depression and suicidal behavior, which revealed decreased CSF levels of kynurenine and increased levels of the neurotoxin quinolinic acid compared to healthy individuals [160]. These findings underscore the kynurenine pathway’s role in neurodegeneration, behavior, and overall neural function [159].

#### 2.2.8. Gut–Brain Axis

Recent studies have highlighted the critical relationship between the gut microbiota and the brain, commonly referred to as the *gut–brain axis* [161,162]. This relationship is bidirectional and mediated through multiple pathways, including the hypothalamic–adrenal axis [163], the autonomic and enteric nervous systems, the immune system [164], the endocrine system, and the gut microbiota and its metabolites [165,166,167,168,169,170]. The latest theories also suggest that bacterial extracellular vesicles may travel to the CNS, exerting their effects there [171].

The gut microbiota plays a vital role not only in maintaining overall host health but also in supporting brain health and development. An imbalance between beneficial and pathogenic bacteria in the gut, known as dysbiosis, can disrupt CNS function [172,173,174]. Dysbiosis has been shown to increase inflammatory cytokines, which alter the permeability of both the blood–brain barrier and the gut barrier, leading to neuroinflammation [175]. Stress further disrupts gut microbiota balance, influencing immune mechanisms and promoting microglial activation and cytokine secretion [176].

This connection is particularly evident in patients with chronic conditions such as inflammatory bowel disease (IBD), who frequently exhibit concurrent MDD [177]. These findings highlight the role of chronic gut inflammation in the development of MDD symptoms [178]. Additionally, a study demonstrated that daily administration of low-dose tricyclic antidepressants produced an 85% moderate response rate in patients with IBD, highlighting the therapeutic potential of targeting the gut–brain axis [179].

Various studies also indicate that probiotics may help alleviate the symptoms in patients with psychiatric disorders, providing further evidence of the gut microbiota’s impact on mental health [180,181,182,183,184,185,186,187,188]. These findings highlight the critical role of the gut–brain axis in the development of inflammation and consequently MDD symptoms, further adding to the mechanisms linking inflammation and depression.

## 3. Results

### 3.1. Anti-Inflammatory Effect of Antidepressants

#### 3.1.1. IL-1β

**Figure 2:**
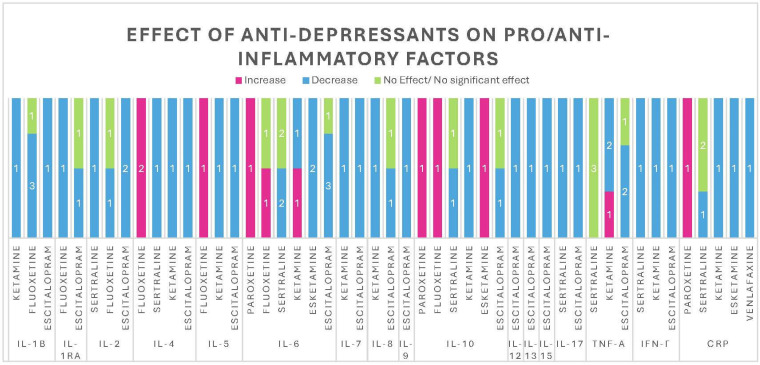
Reported effects of SSRIs, SNRIs, ketamine, and esketamine on cytokine levels. Numbers in the columns indicate how many studies found an increase, decrease, or no effect/no significant effect in cytokine levels after treatment with each type of antidepressant. CRP: C-reactive protein; IL-1RA: IL-1 receptor antagonist; *TNF-α: Tumor Necrosis Factor Alpha; IFN-γ: interferon gamma*.

IL-1β, a cytokine belonging to the IL-1 family, plays a crucial role in the host’s defense against pathogens, particularly in injury and infection, and exhibits potent pro-inflammatory activity [189]. Increased levels of IL-1β have been linked to an increase in Hamilton depression rating scale (HAMD) scores [190]. In our review, we noticed that IL-1β decreased with the administration of the SSRIs escitalopram [191] (one study) and fluoxetine [192,193,194] (three studies) in patients with depression, as well as with ketamine [195] (one study in patients with treatment-resistant depression). However, one study on patients with MDD given fluoxetine showed no significant effect on IL-1β serum levels [196] (Table 1 and Figure 2).

#### 3.1.2. IL-1ra

IL-1ra, also a member of the IL-1 cytokine family, functions as an IL-1 receptor antagonist. By binding to the same receptors as IL-1β and IL-1α, it effectively blocks these cytokines, thereby modulating their pro-inflammatory effects in the human body [197]. Furthermore, studies have shown that it is an effective anti-inflammatory against various diseases such as colitis and arthritis. In our review, we noticed that IL-1ra decreased with the administration of the SSRIs escitalopram [191] and fluoxetine [193] in patients with depression. However, one study on patients with MDD given escitalopram [198] showed no significant effect on IL-1ra serum levels in patients with depression (Table 1 and Figure 2).

#### 3.1.3. IL-2

IL-2 is a cytokine with extensive pro-inflammatory roles, including promoting the differentiation of CD8+ T cells into cytotoxic T lymphocytes [199,200,201] and guiding the maturation of CD4+ naive T cells into regulatory T cells within the thymus [202]. In our review, we noticed that IL-2 decreased with the administration of the SSRIs escitalopram [191,203] (one study in patients with moderate depression, another study in patients with MDD), fluoxetine (one study, patients with MDD) [203], sertraline (one study, patients with unipolar depression) [204], as well as with ketamine (one study, patients with treatment-resistant depression) [195]. However, one study on patients with MDD given fluoxetine showed no significant effect on IL-2 serum levels [205] (Table 1 and Figure 2).

#### 3.1.4. IL-4

IL-4 is a cytokine with immunoregulatory functions, playing a role in modulating inflammation, supporting hematopoiesis, and regulating antibody production [206]. It exerts its anti-inflammatory effect by inhibiting the production of IL-1β and TNF-α [207] and also inducing the production of IL-1ra [208]. In our review, we noticed that IL-4 decreased with the administration of the SSRIs escitalopram (one study, moderate depression) [191] and sertraline (one study, unipolar depression) [204], as well as with ketamine (one study, patients with treatment-resistant depression) [195]. Meanwhile, it increased with the SSRI fluoxetine (two studies, first on depressed patients [193], only at week 8, second on first-episode adolescent patients with moderate to severe major depressive disorder [194]) (Table 1 and Figure 2).

#### 3.1.5. IL-5

IL-5 is a pro-inflammatory cytokine that stimulates the maturation, proliferation, and migration of eosinophils [209,210]. In our review, we noticed that IL-5 decreased with the administration of the SSRIs escitalopram (one study, moderate depression) [191] and with ketamine (one study, treatment-resistant depression) [195]. However, one study [193] classifies IL-5 as an anti-inflammatory cytokine rather than a pro-inflammatory one. Its findings showed an increase in IL-5 levels after administration of the SSRI fluoxetine in depressed patients [193] (Table 1 and Figure 2).

#### 3.1.6. IL-6

IL-6 plays a key role in host defense against pathogens, injury, and infection. Its pro-inflammatory mechanism lies in stimulating immune reactions [211,212], acute-phase responses [213], and hematopoiesis [214]. In our review, we noticed that IL-6 decreased with the administration of the SSRIs escitalopram (three studies, moderate depression [191] and MDD [203,215]), sertraline (three studies, first hemodialysis and depression [216], second unipolar depression [204], and last congestive heart failure and depression [217]), and fluoxetine (two studies, MDD [203] and depression [193]), with ketamine (one study, treatment-resistant depression [195]), with esketamine (two studies, labor and postpartum depression [218], and elective non-cardiac thoracic surgery with high-dose esketamine [219]), while it increased with paroxetine (one study, MDD) [220] and ketamine (one study, MDD) [221].

However, one study on patients with MDD given fluoxetine showed no significant effect on IL-6 serum levels [196]. Another study with escitalopram showed no significant effect on IL-6 serum levels in patients with depression [198]. Two studies with sertraline also showed no effect, the first on patients with MDD and CKD [222] and the second on patients with CHD and comorbid depression [223] (Table 1 and Figure 2).

#### 3.1.7. IL-7

IL-7 has a vital role in various aspects of the immune system, and in inflammation, it acts by promoting pro-inflammatory cells and cytokines [224]. In our review, we noticed that IL-7 decreased with the administration of the SSRI escitalopram (one study, moderate depression [191]) and ketamine (one study, treatment-resistant depression [195]) (Table 1 and Figure 2).

#### 3.1.8. IL-8

IL-8 is a pro-inflammatory chemokine distinguished from other cytokines by its high affinity for signaling and attracting neutrophils [225]. In our review, we noticed that IL-8 decreased with the administration of the SSRI escitalopram (one study, moderate depression [191]) and with ketamine (one study, depression [226]). However, one study on patients with MDD given escitalopram showed no significant effect on IL-8 serum levels in patients with depression [198] (Table 1 and Figure 2).

#### 3.1.9. IL-9

IL-9 is primarily involved in promoting mast cell activity and regulating IgE production [227]; these are linked to the pathogenesis of asthma and protecting the body against parasitic infections [228]. In our review, we noticed that IL-9 decreased with the administration of the SSRI escitalopram (one study, moderate depression) [191] (Table 1 and Figure 2).

#### 3.1.10. IL-10

IL-10 is a cytokine essential for its anti-inflammatory functions, playing a key role in regulating inflammation and preventing autoimmune activation in the body. It works by suppressing the activity of T cells and macrophages [229,230]. In our review, we noticed that IL-10 decreased with the administration of the SSRI escitalopram (one study, moderate depression) [191] and ketamine (one study, treatment-resistant depression) [195], and it increased with the SSRI fluoxetine (one study, Crohn’s disease) [231], paroxetine (one study, MDD) [220], and esketamine (one study, labor and postpartum depression) [218]. However, in another study with sertraline, IL-10 decreased (unipolar depression) [204], and another study on patients with MDD given escitalopram showed no significant effect on IL-10 serum levels [198] and a non-statistically significant increase with sertraline (one study, hemodialysis patients with depression) [216] (Table 1 and Figure 2).

#### 3.1.11. IL-12

IL-12 uniquely regulates inflammation by influencing both the innate and adaptive immune systems. It stimulates T lymphocytes to produce IFN-γ [232] and also supports the cytotoxic activity of T cells and NK cells by promoting them to release perforins and granzymes [233,234,235,236]. In our review, we noticed that IL-12 increased with the administration of the SSRIs escitalopram (one study, moderate depression) [191] and fluoxetine (one study, depressed patients, week 8) [193], while it decreased with fluoxetine (one study, depressed patients, week 4) [193] (Table 1 and Figure 2).

#### 3.1.12. IL-13

IL-13 is primarily an anti-inflammatory cytokine that influences monocytes and B cells, reduces the production of pro-inflammatory cytokines, and induces class switching to IgE [237,238]. These actions contribute to its role in mediating allergic asthma. In our review, we noticed that IL-13 decreased with the administration of the SSRI escitalopram (one study, moderate depression [191]) (Table 1 and Figure 2).

#### 3.1.13. IL-15

IL-15 plays a crucial pro-inflammatory and immunomodulatory role in defending against various pathogens—including viruses, bacteria, and parasites—by activating and stimulating lymphocytes [239]. In our review, we noticed that IL-15 increased with the administration of the SSRI escitalopram (one study, moderate depression [191]), while it decreased with fluoxetine (one study, depressed patients, week 4) [193] (Table 1 and Figure 2).

#### 3.1.14. IL-17

IL-17 is a pro-inflammatory cytokine that promotes the expression of IL-6 and granulocyte colony-stimulating factor (G-CSF), which in turn enhances neutrophil recruitment to the site of infection and stimulates granulopoiesis [240]. In our review, we noticed that IL-17 decreased with the administration of the SSRIs escitalopram (one study, moderate depression [191]) and sertraline (one study, patients with unipolar depression [204]) (Table 1 and Figure 2).

#### 3.1.15. IFN-γ

IFN-γ, produced by T lymphocytes and NK cells, exerts powerful immunomodulatory effects by regulating cell proliferation and apoptosis, as well as activating the innate immune system and macrophages [241,242]. In our review, we noticed that IFN-γ decreased with the administration of the SSRIs escitalopram (one study, moderate depression) [191], sertraline (one study, patients with unipolar depression) [204], and fluoxetine (one study, depressed patients, week 4) [193], as well as ketamine (one study, patients with treatment-resistant depression) [195] (Table 1 and Figure 2).

#### 3.1.16. TNF-α

TNF-α is a cytokine with diverse functions, primarily responsible for promoting inflammation and contributing to the pathogenesis of autoimmune diseases. Additionally, its signaling pathways can lead to cellular apoptosis and necrosis [243,244,245]. In our review, we noticed that TNF-α decreased with the administration of the SSRI escitalopram (two studies, moderate depression [191] and MDD [203]) and ketamine (two different studies on treatment-resistant depression [195,246]). A third study on patients with treatment-resistant depression showed an increase in TNF-α serum levels with ketamine [247]. However, one study on patients with MDD given escitalopram showed no significant effect on TNF-α serum levels in patients with depression [198], and two studies with sertraline also showed no effect (first congestive heart disease and comorbid depression [223], and second on unipolar depression [204]). With the SSRI paroxetine, TNF-a levels were increased in patients with MDD [220] (Table 1 and Figure 2).

#### 3.1.17. CRP

C-reactive protein (CRP) is an acute-phase protein released in response to inflammation and tissue damage [248]. As a result, CRP levels are commonly used to monitor infection, inflammation, and autoimmune diseases [249,250]. In our review, we noticed that CRP decreased with the administration of the SSRI sertraline (one study, congestive heart failure and depression [217]), the SNRI venlafaxine (one study, patients with MDD [220]), ketamine (one study, patients with post-operative cognitive dysfunction after cardiac surgery [251]), and esketamine (one study, labor and postpartum depression [218]). CRP levels were increased in patients with MDD with the SSRI paroxetine [220]. Two studies with sertraline also showed no effect on CRP levels (first on MDD and chronic kidney disease [222], and second on congestive heart disease and comorbid depression [223]) (Table 1 and Figure 2).

**Table 1:** The effect (decrease (↓), increase (↑), or no statistically significant effect (↔)) of antidepressants (SSRIs, SNRIs, ketamine, and esketamine) on inflammatory and non-inflammatory factors; all studies were conducted in serum. *TNF-α: Tumor Necrosis Factor Alpha; NLRP3: Nod-like receptor pyrin-containing 3; IFN-γ: interferon gamma; hsCRP: high-sensitivity C-reactive protein; MDD: major depressive disorder; TRD: treatment-resistant depression; CKD: chronic kidney disease; LPS: lipopolysaccharide; PHA: phytohemagglutinin; CHD: coronary heart disease*.

Name	Class	Effect	Cytokines	Model	Citation
Escitalopram	SSRI	↓	IL-1β, IL-2, IL-5, IL-6, IL-7, IL-8, IL-9, IL-17, TNF-α, IFN-γ	Moderate depression, patients were their own control, serum, Luminex xMAP multiplexing technology, 26 weeks of antidepressant treatment, 90 participants (71% women) with a mean age of 38 years.	[191]
		↓	IL-1ra, IL-4, IL-10, IL-13		[191]
		↑	IL-12, IL-15		[191]
		↔	IL-10, IL-1ra, IL-6, IL-8, TNF-α	Depression, randomized double-blinded trial, whole blood stimulated with LPS or PHA (in vitro), Luminex 100 platform, 4 weeks of antidepressant treatment, 44 participants (28 women), with a mean age of 32.7 years.	[198]
		↓	IL-2, IL-6 and TNF-a	MDD, patients were their own control, cytokines were analyzed in serum by ELISA test, 6 weeks of antidepressant treatment, 65 participants (35 women), with a mean age of 36 years.	[203]
		↓	IL-6	MDD, open-label part-randomized multicenter pharmacogenetic study with two active pharmacological treatment arms, leukocyte mRNA levels were measured, 8 weeks of antidepressant treatment, 38 participants with a mean age of 38 years.	[215]
Fluoxetine		↔	IL-1β, IL-6	MDD, patients were their own control, cytokines were analyzed in serum by ELISA test, 8 weeks of study, 14 participants (10 women), with a mean age of 37 years.	[196]
		↓	IL-2, IL-6 and TNF-a	MDD, patients were their own control, cytokines were analyzed in serum by ELISA test, 6 weeks of study, 65 participants (36 women), with a mean age of 36 years.	[203]
		↑	IL-10	Crohn’s disease, presence of a separate control group that received a placebo, cytokines were analyzed by flow cytometry, 6 months of antidepressant treatment, 26 participants (12 women), with a mean age of 37 years.	[231]
		↓	TNF-a	Depression, patients were their own control, cytokines were analyzed in serum by ELISA test, 12 weeks of antidepressant treatment, 30 participants (20 women), with a mean age of 36 years.	[252]
		↓	IL-1β	Depression, the effect of fluoxetine was compared to the untreated group, cytokines were analyzed in serum by ELISA test, 12 weeks of antidepressant treatment, 32 participants (18 women), with a mean age of 34 years.	[192]
		↔	IL-2, CRP	Depression	[205]
		↓	IL-1ra	Depression, cytokine measurement in the 4th week of therapy, presence of a separate control group, cytokines were analyzed in serum by multiplex bead-based immunoassays, 8 weeks of antidepressant treatment, 22 participants (18 women), with a mean age of 17 years.	[193]
		↓	IFN-γ, IL-1β, TNF-α, IL-6, IL-12, IL-15		[193]
		↑	IL-4	Depression, cytokine measurement in the 8th week of therapy.	[193]
		↑	IL-12 and IL-5		[193]
		↔	IL-10 and IL-13	Depression	[193]
		↓	CRP	COVID-19; during a mid-hospital stay, a double-blind randomized, placebo-controlled clinical trial; 4 weeks of antidepressant treatment, 72 participants (35 women), with a mean age of 52 years.	[253]
		↓	NLRP3, IL-1β, and IL-18	First episode, moderate to severe MDD, patients were their own control, cytokines were analyzed in serum by ELISA test, 12 weeks of antidepressant treatment, 48 participants (30 women), with a mean age of 17 years.	[194]
		↑	IL-4		[194]
Sertraline		↔	hsCRP, IL-6	MDD and CKD, randomized, double-blind placebo-controlled trial, cytokines were analyzed in serum by ELISA test, 12 weeks of antidepressant treatment, 201 participants (61 women), with a mean age of 58 years.	[222]
		↓	IL-6	Hemodialysis and depression, randomized double-blind, placebo-controlled clinical trial, cytokines were analyzed in serum by ELISA test, 12 weeks of antidepressant treatment, 43 participants (16 women), with a mean age of 63 years.	[216]
		↔	TNF-α		[216]
		↔	IL-10		[216]
		↓	IL-4, IL-10	Unipolar depression, double-blind, placebo-controlled trial, blood cytokines were measured by flow cytometry, 6 weeks of antidepressant treatment, 120 participants (82 women), with a mean age of 42 years.	[204]
		↓	IL-2, IL-6, IL-17a, IFN-γ		[204]
		↔	TNF-α		[204]
		↔	hsCRP, IL-6, and TNF-α	CHD and MDD, randomized, double-blind, placebo-controlled trial, cytokines were analyzed in serum by ELISA test, 10 weeks of antidepressant treatment, 122 participants (41 women), with a mean age of 59 years.	[223]
		↓	CRP and IL-6	CHD and MDD, randomized double-blind, placebo-controlled clinical trial, cytokines were analyzed in serum by ELISA test, 20 weeks of antidepressant treatment, 95 participants (48 women), with a mean age of 57 years.	[217]
Paroxetine		↑	IL-10	MDD, two randomized placebo-controlled clinical studies, cytokines were analyzed in serum by ELISA test, 10 weeks of antidepressant treatment, 106 participants (72 women), with a mean age of 46 years.	[220]
		↑	TNF-α, IL-6 and CRP		[220]
Venlafaxine	SNRI	↓	CRP	MDD, two randomized placebo-controlled clinical studies, cytokines were analyzed in serum by ELISA test, 10 weeks of antidepressant treatment, 104 participants (64 women), with a mean age of 45 years.	[220]
Ketamine		↓	CRP	Post-operative cognitive dysfunction, patients randomly received placebo or an i.v. bolus of ketamine (0.5 mg/kg) during anesthetic induction. Anesthesia was maintained with isoflurane and fentanyl. A nonsurgical group was also included as control, serum C-reactive protein (CRP) concentrations were determined before surgery and on the first post-operative day, 96 participants with a mean age of 66 years.	[251]
		↓	IL-8/IL-10 ratio	MDD, presence of a separate control group that received a placebo, plasma concentrations of cytokines were analyzed by ELISA test 24 h after ketamine infusion (0.5 mg/kg), 25 participants (7 women) with a mean age of 46 years.	[254]
		↓	IL-4, and IL-10	TRD, cytokines were analyzed in plasma by multiplex bead-based immunoassays on days 13 and 26, TRD patients received intravenous ketamine (0.5 mg/kg) three times weekly for 2 weeks, 66 participants (37 women) with a mean age of 36 years.	[195]
		↓	IFN-γ, IL-17α, IL-1β, IL-2, IL-23, IL-5, IL-6, IL-7, and TNF-α		[195]
		↓	IL-8	Depression, presence of a separate control group that received a placebo, depressed patients (n = 46, female, n = 17), cytokines were analyzed in plasma by multiplex bead-based immunoassays 24 h after infusion of ketamine (0.5 mg/kg).	[226]
		↓	TNF-α	TRD, randomized, double-blind control study, patients were randomized into three groups according to the treatment received: 0.5 mg/kg ketamine, 0.2 mg/kg ketamine, and normal saline infusion, cytokines were analyzed in plasma by ELISA test at baseline and at 40 min, 240 min, day 3, and day 7 post-infusion, 71 participants (53 women), with a mean age of 46 years.	[246]
		↑	IL-6	MDD, double-blind, placebo-controlled studies, cytokines were analyzed in plasma by multiplex bead-based immunoassays, blood samples were received at 60 min before ketamine infusion and at 230 min, one day, and three days post-infusion ketamine (0.5 mg/kg) or saline placebo, 80 participants (61 women), with a mean age of 45 years.	[221]
		↑	TNF-α	TRD, randomized placebo-controlled and open-label trials, cytokines were analyzed in plasma by ELISA test, 78 patients were allocated to receive two ketamine infusions (n = 30, days 1 and 4), a single ketamine (0.5 mg/kg) infusion (n = 24, only day 1), or normal saline placebo infusion (n = 24, only day 1).	[247]
Esketamine		↓	IL-6 and CRP	Labor and postpartum depression, a randomized, double-blinded controlled trial, cytokines were analyzed in plasma by ELISA test, a total of 120 women who underwent labor analgesia by epidural analgesia pump were enrolled and divided into two groups randomly. Esketamine at a dose of 0.2 mg/kg was intravenously injected after fetal disengagement in the test group and placebo was administered in the control group.	[218]
		↑	IL-10		[218]
		↓	IL-6	Elective non-cardiac thoracic surgery under general anesthesia, randomized controlled trial. During the operation, patients received 0.2 mg/kg (low-esketamine group) or 0.5 mg/kg esketamine (high-esketamine group) vs. placebo, cytokines were analyzed in plasma by ELISA test before surgery, post-operative day 1 and day 3, 129 participants (56 women), with a mean age of 65 years.	[219]

## 4. Discussion

Major depressive disorder (MDD) is the most common mental health condition worldwide, yet its underlying pathophysiology remains unclear. The monoamine hypothesis, which attributes depression to deficits in neurotransmitters like serotonin, norepinephrine, and dopamine, has traditionally shaped the development of antidepressants aimed at restoring monoamine balance in the synaptic cleft [14,23]. However, the prevalence of treatment-resistant depression and growing evidence from recent studies have led researchers to explore alternative mechanisms, including the potential role of inflammation and inflammatory markers in the development of depression [255,256,257]. These effects are mediated through multiple inflammatory pathways, including NF-κB, the NLRP3 inflammasome complex, the glutamatergic system, the gut–brain axis, the HPA axis impairing neuroplasticity, and the kynurenine pathway [74,75,76,77]. As this can be a very important avenue to be investigated in the pathophysiology of depression, we set out to combine all available human studies on the effects of antidepressants, specifically SSRIs, SNRIs, esketamine, and ketamine, on pro-inflammatory and anti-inflammatory markers, to see if there is a clear link between antidepressant treatment and a decrease in inflammation in the body.

### 4.1. Mechanisms of Anti-Inflammatory Action

The exact mechanism behind the anti-inflammatory effects of antidepressants remains unclear, though several theories have been proposed. It is known that ketamine exerts anti-inflammatory action through the NF-κB pathway [258]. Similarly, some evidence suggests that SSRIs and SNRIs may influence the NF-κB pathway and exert anti-inflammatory effects through the NLRP3 inflammasome complex [259].

#### 4.1.1. NF-κB

The transcription factor NF-κB plays a central role in regulating both the innate and adaptive immune systems and acts as a key mediator of inflammation in the body [260]. It promotes the synthesis and release of pro-inflammatory cytokines [261] and has been found to be elevated in patients with depression [262]. There are two pathways of NF-κB activation in the CNS, the classical canonical pathway and the alternative non-canonical pathway [258], and they are activated by a variety of immune- and stress-related stimuli [263]. The canonical pathway is activated by various stimuli, including reactive oxygen species (ROS) [264], pathogen-associated molecular patterns (PAMPs), cytokines (TNF-α, TNF-β, IL-1), microbial components, and many other factors [263,265]. These triggers activate the IκB kinase complex (IKK), which phosphorylates the inhibitory IκB proteins, marking them for ubiquitination and subsequent degradation by the proteasome. This degradation releases the NF-κB dimers, allowing them to migrate into the nucleus and initiate the transcription of target genes [265,266,267,268].

Alternatively to the canonical pathway, the non-canonical pathway is only activated by specific stimuli, including the CD40 ligand, neurotrophic factors, lipopolysaccharide (LPS), and lymphotoxin β [267,269]. Furthermore, it does not rely on the IKK complex but instead is mediated by the NF-κB-inducing kinase (NIK), which activates IKKα [270,271,272]. This complex phosphorylates the NF-κB2 precursor protein, p100, marking it for ubiquitination and degradation by the proteasome. The resulting active complex translocates to the nucleus [267,269,270,271].

The NF-κB pathway has been suggested as a potential target for ketamine. Recent studies indicate that ketamine exerts a suppressive effect on NF-κB signaling in depression and inflammation models, leading to a reduction in levels of stress mediators and pro-inflammatory factors [258]. Reduced levels of NF-κB have been observed in various brain regions in animal studies [258] as well as in blood cells, further supporting this connection [273,274,275,276]. Furthermore, these studies have also shown that there is a potential link between NF-κB and BDNF, which could explain the anti-inflammatory and antidepressant effects of ketamine [258,277]. To date, only one clinical trial has investigated the effects of ketamine on NF-κB-mediated inflammation. Conducted in patients with acute lung injury induced by mechanical ventilation, the study demonstrated that ketamine administration significantly reduced serum levels of NF-κB and other inflammatory markers [278]. Ketamine inhibited NF-κB activity, as evidenced by decreased phosphorylation of the p65 subunit, and was associated with lower levels of oxidative stress markers and pro-inflammatory cytokines. These findings suggest that ketamine’s anti-inflammatory properties may involve suppression of NF-κB signaling [258].

Additionally, the modulation of the NF-κB signaling pathway by SSRIs and SNRIs has been investigated, with fluoxetine being the most extensively studied for its anti-inflammatory effects. Under ischemia/reperfusion conditions, fluoxetine decreased NF-κB activity by binding to IκB-α, thereby preventing its degradation and leading to increased IκB-α levels in BV-2 microglial cells [279]. Another study using a model of human hippocampal progenitor cells in an inflammatory environment demonstrated that treatment with venlafaxine and sertraline was associated with a decrease in NF-kB activity [280].

#### 4.1.2. NLRP3 Inflammasome Complex

The Nod-like receptor protein 3 (NLRP3) inflammasome complex plays a central role in the innate immune response, providing protection against viral, bacterial, and fungal pathogens [281,282,283,284]. Once activated, the inflammasome can effectively mediate inflammation and initiate a robust immune response.

Recent studies have shown that activation of the NLRP3 inflammasome complex plays a role in the mediation of depression [259]. In mice subjected to chronic unpredictable mild stress (CUMS), which has been shown to yield the same effect as depression [285], levels of NLRP3 were increased in hippocampal regions [286,287]. Postmortem brain and serum analyses from patients with MDD have demonstrated activation of the NLRP3 inflammasome [288,289,290]. Supporting this observation, recent studies show that chronic antidepressant administration exerts anti-inflammatory effects, particularly within the hippocampus, suggesting that inhibition of NLRP3 inflammasome pathways may represent a potential mechanism underlying the antidepressant efficacy of drugs [259,291]. Ketamine has also been shown to exert antidepressant effects through inhibition of the NLRP3 inflammasome, as demonstrated in a recent study in a mouse model [292]. Furthermore, direct suppression of NLRP3 inflammasome activation by various inhibitors has been proposed as a promising therapeutic strategy for depression [293].

#### 4.1.3. SSRIs

Due to the predominance of the monoamine theory of depression, SSRIs and SNRIs remain the most widely prescribed medications for the treatment of depression and are the first-line pharmacological interventions recommended in clinical guidelines [294]. Consequently, these drug classes have been the most extensively studied in relation to their effects on both inflammatory and non-inflammatory factors.

##### Fluoxetine

Fluoxetine was the first SSRI approved for the treatment of MDD in the US market [295,296]. Since its introduction, depression response rates have been between 60 and 70% with the various antidepressants that followed [297,298]. Fluoxetine is widely prescribed by physicians due to compelling characteristics such as low side effects, simple one-per-day dosing, and overdose safety. These factors contribute to better patient compliance and improved treatment outcomes [295].

Analyzing our results, we find that fluoxetine had the most studies on its effectiveness as an antidepressant. It was also found to primarily decrease the levels of most pro-inflammatory cytokines (IL-1β, IL-2, IL-6, IL-12, IL-15, NLRP3, IL-18, IFN-γ, TNF-α) in the studies [192,193,194,203,252,253] and increase the level of anti-inflammatory cytokines IL-4 [193,194] and IL-10 [231] (Table 1 and Figure 2). These results support our hypothesis that the administration of antidepressants can decrease pro-inflammatory factors and increase anti-inflammatory factors, which will overall result in a decrease in inflammation in the body.

Contradictory results to our hypothesis come from the same study, which found an increase in the pro-inflammatory factors IL-12 and IL-5 at week 8, and a decrease in the anti-inflammatory cytokine IL-1ra at week 4 which was then later restored at week 8, and no significant difference in clinical follow-up for anti-inflammatory cytokines IL-10 and IL-13; the reason for this is not explained by the authors [193]. Finally, two studies [196,205] found no significant effect on the pro-inflammatory factors IL-1β, IL-2, IL-6, and CRP (Table 1 and Figure 2).

##### Escitalopram

Escitalopram is characterized by being a potent and the most selective SSRI; it acts by specifically inhibiting SERT and consequently increasing serotonin levels in the CNS [299]. Pro-inflammatory factors (IL-1β, IL-1ra, IL-2, IL-4, IL-5, IL-6, IL-7, IL-8, IL-9, IL-17, TNF-α, IFN-γ) were decreased across studies [191,203,215], with one study [198] noting no statistically significant effects on IL-10, IL-1ra, IL-6, IL-8, and TNF-α (Table 1 and Figure 2).

On the other hand, one study [191] showed that anti-inflammatory factors (IL-1ra, IL-10, IL-13) were decreased and pro-inflammatory factors (IL-12 and IL-15) were increased; this was not explained by the authors of the study (Table 1 and Figure 2).

A common pattern observed with the SSRIs fluoxetine and escitalopram is a decrease in the anti-inflammatory cytokine IL-1ra and an increase in the pro-inflammatory cytokine IL-12 following their administration.

##### Sertraline

Sertraline is a widely used SSRI that is effective for treating depression [300]. Furthermore, research has shown that sertraline outperforms other antidepressants, demonstrating greater efficacy than fluoxetine and better tolerability compared to amitriptyline, imipramine, paroxetine, and mirtazapine [300,301]. This could possibly explain why it has the most consistent results to support our hypothesis, as it has no contradictory increase in pro-inflammatory factors with the administration of sertraline. Pro-inflammatory factors (IL-2, IL-4, IL-6, IL-10, IL-17a, IFN-γ, TNF-α, and CRP) were decreased across studies [204,216,217], with three studies [204,222,223] noting no statistically significant effects on CRP, IL-6, and TNF-α. Additionally, an increase in the anti-inflammatory cytokine IL-10 was found in one study, although it was also not found to be statistically significant [216]. Moreover, a decrease in anti-inflammatory cytokines IL-4 and IL-10 was found in the same study [204] (Table 1 and Figure 2).

##### Paroxetine

Paroxetine is a well-tolerated SSRI with a low side effect profile and is effective in the treatment of MDD. It was also found to be effective in preventing relapses in patients treated for up to one year [302]. In the study investigated, an increase in the anti-inflammatory cytokine IL-10 and a contradictory increase in CRP were found [220]. A common pattern observed is an increase in the pro-inflammatory factors TNF-α and IL-6 in both paroxetine [220] and ketamine [221,247] (Table 1 and Figure 2). There are not enough current studies that follow our exclusion criteria to be able to draw any conclusions on the overall effect of paroxetine and fluvoxamine on anti-inflammation.

#### 4.1.4. SNRIs: Venlafaxine

SNRIs were initially developed for patients with depression who did not respond to SSRIs, offering a treatment option with fewer side effects than tricyclic antidepressants. Studies have shown that at higher doses, venlafaxine is effective in treating resistant depression, with a side effect profile comparable to that of SSRIs [303]. Only CRP was studied in the article on venlafaxine, and its levels were decreased after treatment with it in patients with MDD [220] (Table 1 and Figure 2).

#### 4.1.5. SSRIs vs. SNRIs

In conclusion, both SSRIs and SNRIs are found to support our hypothesis that antidepressants have an anti-inflammatory effect, and this is by decreasing levels of pro-inflammatory cytokines and increasing levels of anti-inflammatory factors. There are a few exceptions to some studies not following the same pattern and some showing no significant effects.

#### 4.1.6. Ketamine/Esketamine

Ketamine has long been used as general anesthetic; however, recent studies have begun to explore its effectiveness in treating treatment-resistant depression [258,304,305,306]. Its mechanism of action primarily involves antagonism of the NMDA receptor and modulation of the NF-κB pathway, making its effects on depression and inflammation independent of the traditional monoamine hypothesis [258]. The use of ketamine for depression treatment remains a relatively new area of research, and its full therapeutic potential is still being investigated across various studies [304]. Pro-inflammatory factors (CRP, TNF-*α*, IL-6, IL-8/IL-10 ratio, IFN-γ, IL-17α, IL-1β, IL-2, IL-5, IL-6, IL-7) were decreased across studies [218,226,246,251,254]. Two studies investigating IL-6 and TNF-α separately showed them to increase upon administration of ketamine [221,247]. The authors of the former study concluded that post-infusion cytokine levels were not correlated with the antidepressant response to ketamine. Notably, the latter study observed an increase in TNF-α levels after two infusions of ketamine; alternatively, a single ketamine infusion was found to improve the TNF-α-to-IL-2 ratio when compared to either two ketamine infusions or a single placebo infusion [247]. Moreover, another study reported a decrease in anti-inflammatory cytokines IL-4 and IL-10 following ketamine administration [195].

Esketamine, a stereoisomer of ketamine, not only exhibits more potent anesthetic properties but has also shown effectiveness in treating individuals with severe, treatment-resistant depression [307]. It is currently the only NMDA receptor antagonist approved by the FDA and the European Medicines Agency (EMA) for this indication [42,307]. Both studies investigating esketamine reported a reduction in the pro-inflammatory cytokine IL-6, one in the context of labor and postpartum depression, and the other in patients undergoing elective non-cardiac thoracic surgery [218,219]. However, in the latter study, this effect was observed only with high-dose esketamine, as the low-dose regimen did not produce a significant change. Additionally, the former study also noted a decrease in C-reactive protein (CRP) levels and an increase in the anti-inflammatory cytokine IL-10, findings that are consistent with our hypothesis [218] (Table 1 and Figure 2). Given esketamine’s recent introduction into clinical practice, further research is warranted not only to thoroughly assess its long-term safety and therapeutic efficacy [307] but also to explore its potential immunomodulatory effects. Moreover, current studies investigating the relationship between ketamine and inflammation are limited; however, the existing evidence is highly promising and largely supportive of our hypothesis.

### 4.2. Limitations of Evidence

Comparing the results of these studies is challenging due to numerous limitations in measurement methods, reporting standards, and study settings. The studies reviewed exhibit considerable variability in sample sizes, ranging from as few as 22 participants to larger sample sizes of 73 [204], 90 [191], and 122 [223]. This variation introduces challenges in drawing generalizable conclusions. Additionally, the studies involve diverse age groups of adolescents [193], adults [191,204], and elderly people [223], which may further influence the outcomes and complicate cross-study comparisons. Differences in study duration also contribute to inconsistencies, with some studies spanning over 1 to 3 days [221], others extending to 4- and 8-week follow-ups [193], and some over 8-, 12-, and 26-week follow-ups [191].

Placebo-controlled trials are critical for ensuring the validity and interpretability of study outcomes. For example, one study reported a decrease in pro-inflammatory cytokines; however, similar decreases were observed in the placebo group [204]. This highlights the need for well-designed control conditions to accurately understand the effects of the treatment. Moreover, a common difference found across studies was the presence or absence of a control or placebo group. While the majority of studies included these groups [192,193,194,195,196,198,203,204,205,215,216,217,218,219,220,221,222,223,231,246,247,251,253,254,308], a few [191,226,252] did not, and instead measured only the changes over time within the same group of participants. This lack of a control group limits the ability to attribute observed changes to the intervention itself, thereby reducing the reproducibility and interpretive clarity of the findings, which can reduce the strength and reliability of the study’s conclusions. However, only 3 out of the 26 studies included in our review lacked a control group, representing a relatively small proportion of the total. Properly designed control and placebo groups are essential to ensure that observed effects can be confidently attributed to the intervention rather than to external or nonspecific influences. This enhances both the internal validity of the studies and the strength of their conclusions, thereby supporting more reliable scientific interpretations and future research directions in the field.

Another limitation of this review lies in the strict inclusion and exclusion criteria. Specifically, only studies that assessed inflammatory changes through cytokine measurement and simultaneously explored a connection with depression were included. While this approach ensured a focused analysis, it significantly reduced the number of eligible studies, potentially limiting the scope and generalizability of the findings. As a result, the conclusions drawn may not fully represent the wider body of existing literature on the subject, particularly studies employing alternative biomarkers or methodological frameworks.

## 5. Materials and Methods

### 5.1. Literature Search

A literature search was carried out in PubMed databases starting July 2024, using the queries ((escitalopram) or (esc))/(fluoxetine)/(Sertraline)/(Fluvoxamine)/(Paroxetine)/(Duloxetine)/(Venlafaxine)/(Ketamine)/(Esketamine) and (anti-inflammatory) or (inflammation) or (pro-inflammatory)) and ((depression) or (antidepressant)) Filters: Clinical Trial, Randomized Controlled Trial, from 2009 to 2024.

**Figure 3:**
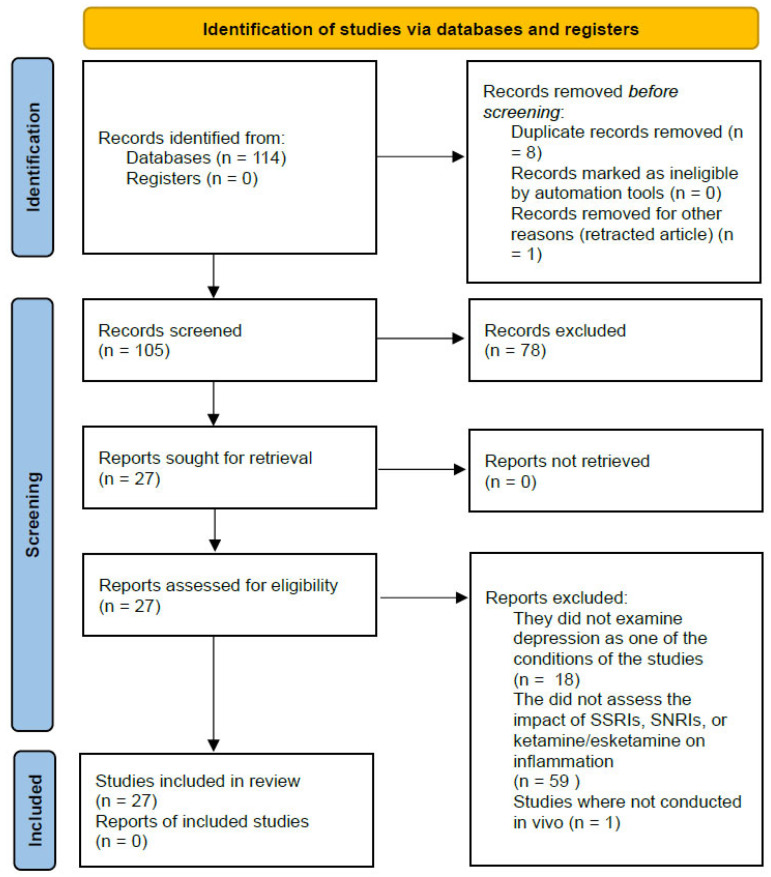
The Preferred Reporting Items for Systematic Reviews and Meta-Analyses (PRISMA) [309] flowchart.

The Preferred Reporting Items for Systematic Reviews and Meta-Analyses (PRISMA) flowchart is presented in Figure 3.

### 5.2. Eligibility Criteria

Not all studies identified through the search filter were included in this review. Despite the specificity of the search criteria, some retrieved studies did not meet the inclusion criteria, e.g., they did not examine depression as one of the conditions of the studies, or assess the impact of SSRIs, SNRIs, esketamine, or ketamine on inflammation; moreover, some studies were not conducted in vivo. Each search result was carefully screened, and only studies that explicitly addressed these key variables were selected. As a result, a significantly smaller number of studies were included after a thorough review of all retrieved articles.

A total of 114 studies were initially screened. Following the exclusion of duplicates and selection based on intervention, relevance, and study design, 27 studies met the eligibility criteria and were included in the analysis. Their results are discussed in the text and presented in Table 1.

## 6. Conclusions

The relationship between inflammation and depression is complex and remains an area of active investigation. Despite the numerous variables in the studies presented in this review, which are discussed in the limitations section, and the application of strict inclusion and exclusion criteria, it seems likely that there is a link between antidepressants and their anti-inflammatory effects. Levels of pro-inflammatory factors were mostly found to be decreased with treatment with SSRIs (escitalopram, fluoxetine, sertraline, paroxetine, and fluvoxamine), SNRIs (venlafaxine), esketamine, and ketamine. Additionally, the anti-inflammatory cytokine IL-10 was found to be increased upon treatment with fluoxetine, paroxetine, and esketamine, and IL-4 was increased upon treatment with fluoxetine. However, it should be emphasized that there are studies showing effects different from those described, which means that in this conclusion, we are only pointing out certain patterns and associations between antidepressant drug classes and changes in cytokine levels. This presents a new potential for investigating the pathophysiology of depression as it presents more evidence of its relationship with inflammation and how the treatment of inflammation could decrease depressive symptoms. Such findings can also provide insight into new therapeutic approaches for MDD. Further research is necessary to gain a more comprehensive understanding of the relationship between antidepressant therapy, inflammatory pathways, and treatment outcomes in depression. Long-term and larger-scale studies are needed to determine whether current findings reflect only acute effects or are sustained over time, and to evaluate the reproducibility and broader physiological impact of antidepressants. Additionally, (i) randomized controlled trials (RCTs) stratified by demographic factors such as age and gender are necessary to determine how these variables influence the inflammatory response and therapeutic outcomes. Incorporating pre-treatment and post-treatment inflammatory biomarker profiling would help elucidate differential responses across subgroups. Moreover, (ii) studies investigating specific molecular markers within inflammatory pathways, such as NF-κB and the NLRP3 inflammasome, may help identify predictors of therapeutic efficacy. Finally, given the limited existing evidence, (iii) further clinical trials exploring the anti-inflammatory and antidepressant effects of novel agents such as esketamine are also warranted.

## Data Availability

No new data were created or analyzed in this study. Data sharing is not applicable to this article.
